# Hospital Progress and Finance

**Published:** 1923-11

**Authors:** 


### HOSPITAL PROGRESS AND FINANCE.

### FIRES IN HOSPITALS.

FIRES in hospital are chiefly useful for the hitherto neglected lessons that they enforce, and thus it is with two of the most recent and alarming. When a block of the hospital for disabled soldiers in the Midlands was destroyed the day before the Prince of Wales visited its neighbourhood, and thus led him aside to condole with the escaped and injured patients, the lesson unmistakably was that, if disabled men, whether civilians or soldiers, are to be housed in temporary buildings, these last must be fireproof, a condition that the Ministry of Health should enforce without exception. It needed " the tonic touch of a great misfortune," the grievous suffering to which the victims of this fire had to be abandoned, to drive this elementary lesson home. The recommendations resulting from the inquiry into the Harborne fire are as practical as could have been expected, but there can be no real safety where wooden huts are concerned. In regard to the explosion that still more recently wrecked one of the theatres at King's College Hospital, the lesson clearly was that the frames of the cylinders, whose contents presumably expanded to bursting point by the great heat of those summer weeks, should be of metal not of wood, even though, perhaps, at such a temperature no precaution is to be guaranteed. King's College Hospital has already decided, we understand, to make this change.

### THE LISTER WARD: AN ARCHITECT'S CONCLUSION.

THE fate of the famous Lister Ward in Glasgow Royal Infirmary has been the subject of so much controversy (indeed of contradictory " decisions ") that the public may be excused for not remembering what is still intended. Very timely, therefore, is the temperate argument advanced by Mr. James A. Morris, F.R.I.B.A., called " A Humble Plea," for its retention that Messrs. Maclehose, Jackson & Co. have issued in a sixpenny pamphlet. The onus of proof lies with those who would destroy this National Monument in the supposed interests of the Infirmary. They have to show cause why destruction is required, since tradition, good sense, the interest of Europe and America, medical and lay, is opposed to it. The sentimental argument, which is never of greater weight than in regard to hospitals, needs no emphasis. It is all but overwhelming. Only the practical effect of retention on the Infirmary's work needs to be considered, and here Mr. Morris has come to the rescue with his plans and illustrations. The would-be destroyers, he reminds us, allege that the Ward may be septic; that the space it fills would provide essential light and air; that the symmetry of the two sides of the forecourt would suffer; and, ironically enough, the contradictory suggestion is also implied that the site might be wanted for other buildings! But, against these, the Ward is now used as a cloak-room for students, who, so far, have not been decimated by disease. The historic Ward is some eighteen feet high, and the nearest block some sixty feet distant; its modest height could not affect the sky-line of the Infirmary buildings, which the top storeys of the old block do. Therefore, Mr. Morris contends, in effect, why cut down a flower when the crest of the tree above it is causing the trouble ?

### A DECISIVE ARGUMENT.

THE Lister Ward is on the ground floor, and to appreciate the force of this contention it is sufficient to glance at the plans, which are the decisive argument, in reality, as in this book. The symmetry of the forecourt, in which the Ward occupies a small peninsula, is not worth purchasing at the sacrifice of this historic Ward. Indeed, mechanical symmetry is a heresy characteristic of recent architecture, which the great architects of the past, except in ancient Greece, have always contrived to break or disguise. Mr. Morris concludes with a brief explanation how the structural problem of retaining the ground floor Ward could technically be met at an estimated cost of £1,800, a sum that could probably be raised (even in Glasgow itself!) since all authorities worth considering, except some on the board of managers, are in favour of retaining the Lister Ward on any reasonable terms. It is still not too late to withhold a step that Glasgow will live to repent if she is rash enough to take it. The present board are but the trustees of posterity, and posterity will never forgive such vandalism as is contemplated. To commit it would be worse than wanton, for an unnecessary act is doubly wrong, and Mr. Morris has done a public service by his temperate contribution to the discussion, of which, we hope, the commission in charge of national memorials, the Scottish division of H.M. Office of Works, the Society for the Protection of Ancient Buildings, and the Architecture Club will not fail to take note.

### A SUGGESTIVE CRITICISM.

MR. H. CROUCH, Secretary of the Somerset Committee, has read an interesting paper to a meeting of representatives of voluntary hospitals in the Bristol, Somerset, and Wiltshire areas. Mr. Crouch urged co-operation between the Bristol Committee and the committees of neighbouring counties. The Somerset Committee's report had shown that county not to form a suitable unit for a Local Committee, for Taunton was an independent hospital area. Bath and Bristol were equally so. In regard to workmen's contributions, Mr. Crouch added, the Hospitals first in this field have reaped the most. "There has been no attempt to assess the levy on actuarial calculations, or according to the requirements of the hospitals, or to the benefits received by the contributor." He then moved and carried a resolution to appoint a joint committee, consisting of four members of each existing committee, to make recommendations toward greater co-operation among the hospitals concerned, in town and country. Mr. Crouch's interesting criticism of the haphazard way in which even workmen's contributions are collected in competition, without full regard to the work and needs of the hospitals inviting them, deserves careful reflection. He supported it by telling examples, and the moral is that the largest sums are received by the best organised appeal departments. A united hospital council, such as already exists in several large towns, would solve the difficulty. But can this principle be applied to western county areas ?

### PRICE OF POOR LAW RELIEF.

A HEALTHY state of panic exists over the disqualifications legally imposed upon the sick who take the Ministry of Health's advice to enter the paying wards of a Poor Law Infirmary. Now that a pensioner has thereby been legally deprived of his pension, and a Guardian has lost his seat upon the Board, the poor law stigma seems more alive and pernicious than ever. To stay the revolt, without removing its cause, assurances have been given that voters do not necessarily lose their votes if and when they become paying patients in an infirmary. This, however, is a very minor aspect of the question, with which we need not deal at length, since the grave anomaly remains at present unremedied. The Ministry of Health has been placed in so awkward a position by the working of the present law that it can hardly remain, or wish to remain, with folded hands. Nor can the hospitals with a clear conscience assure the unfortunate patients upon their heavy waiting lists that there is room and excellent treatment to be had at the infirmaries, unless they remind them of the heavy price which has to be paid for it. There is no solution but for the Ministry, the Guardians, the electorate and public opinion to press unremittingly for the alteration of the law.

### THE VOLUNTARY SYSTEM IN AUSTRALIA.

THE Conference convened in the summer on the hospital problem of Australia decided almost unanimously to retain the voluntary system on a reformed basis and with central control. A detailed account appeared in the Prince Alfred Hospital *Gazette*, of Sydney, which is interesting also to Englishmen. The pound-for-pound system by which the State has subsidised voluntary funds has been felt to need improvement, but the situation is very like our own, and, in all departments, co-ordination seems to be the remedy. In Australia two systems obtain—reliance on State finance and control in South Australia, Western Australia, and Tasmania, and the quasi-voluntary system adopted in Australia's three largest States—New South Wales, Queensland, and Victoria—where the public contributes one-half or more of the hospitals' whole income. The conclusions of the conference, noticed above, are expected to affect the Hospital Bill that Mr. Oakes has promised to introduce. Briefly, the scheme adopted is expected to abrogate the former pound-for-pound scheme; to retain the quasi-voluntary system, with honorary medical staffs; to secure Government support on the basis of work done with due regard to contributions received from the public, from patients, local bodies, industrial organisations and friendly societies. The scheme, further, would be controlled by a central body, and, judged by the Minister's remarks, would be free from political or official control. In sum, the voluntary system is likely to remain the inspiration and chief stay of the hospitals in the Dominion.

### HOSPITAL CHAIRMAN ON NURSES.

A HOSPITAL chairman recently discussed the nursing situation as it affects hospitals. The hospital, he said, since the war had doubled the salaries of nurses, and doubled their monthly leave. The College of Nursing had asked the hospitals not to continue to offer higher salaries. The general dearth of suitable probationers was due to the want of the right kind of candidate, who did not now seek nursing work. Higher salaries were no remedy for this, because the right kind of candidate was one who came forward intent rather to give than to get. These, or many of them, were now entering the medical profession, who would formerly have entered the hospitals as probationers. Those with the capital became women doctors. The situation is serious and suggests, in no invidious spirit, a possible future for the male nurse. He has been to some extent accidentally restricted. The male cook and the male "housemaid" earn golden opinions when they can be found. There are many men of the right kind, without capital, whom the conditions of peace since 1918 might send into nursing, as did the previous four years send women on to the land and into the war. No doubt such a revolution would demand many changes. But we live in a time of change, and since the shortage of probationers cannot continue indefinitely without producing its own remedy, however strange, we must be prepared to consider the possibility of the male probationer. Many ex-war nurses declare the scarcity of probationers to be due to the autocratic conduct of the matrons and sisters; the effect of this has been to convert their war staffs into women who warn all potential nurses to shun nursing at all costs.

### AN ORTHOPÆDIC HOSPITAL FOR YORKSHIRE?

A CONFERENCE of bodies interested in child welfare has been held at York, when General H. R. Mends, County Director of Infant Welfare Work, outlined a scheme to form a local committee to arouse sympathy for the proposed orthopædic hospital for children and their concurrent education. In the three Ridings are some 4,000 crippled children of school age, of whom three-quarters are considered curable by early and prolonged treatment. For these at present only 250 beds are available. Hence the need for a special hospital where treatment and education can be provided for them. It is thought that the cost would be mainly for capital expenditure on premises and equipment, and General Mends expects that Government grants will largely cover maintenance and educational expenses. The scarcity of beds, which is the main defect of the voluntary system, applies to special no less than to general hospitals, and the case for such a hospital, of which the benefits have been abundantly proved elsewhere, is all the stronger if no great fears are aroused by the problem of its maintenance. In the circumstances therefore, we trust that the project will be loyally supported, and that, in the interests of economy alone, the money will be forthcoming to remedy this defect in Yorkshire's supply of institutions.

